# Safety and efficacy of low-dose esketamine in laparoscopic cholecystectomy: a prospective, double-blind randomized controlled trial

**DOI:** 10.1186/s12871-024-02429-5

**Published:** 2024-02-01

**Authors:** Lu Zhao, Zhengyu Li, Bi Jin, Nina Hou, Heng Yang

**Affiliations:** Department of Anesthesiology, The Third Affiliated Hospital of Anhui Medical University, The First People’s Hospital of Hefei, Hefei, 230061 Anhui China

**Keywords:** Esketamine, Laparoscopic gallbladder surgery, Stress and inflammatory response, Cognitive function

## Abstract

**Background:**

Esketamine, recognized for its analgesic, sedative, and anti-inflammatory qualities, is integral in multimodal analgesia. However, the potential opioid-sparing effects of intravenous esketamine, along with its impact on inflammatory responses, and cognitive function during laparoscopic surgery, remain unexplored.

**Methods:**

In this study, 90 patients scheduled for laparoscopic cholecystectomy were equally randomized into three groups: a normal saline control group (NS), a low-dose esketamine group (LS) and a high-dose esketamine group (HS). Subsequently, we monitored several parameters: hemodynamics, levels of stress and inflammatory responses, intraoperative doses of sufentanil, remifentanil, and propofol, and 24-hour postoperative sufentanil requirements. We also evaluated alterations in cognitive function, perioperative indicators, and potential adverse reactions among the three groups.

**Results:**

Compared to their levels 5 minutes prior to anesthesia (T_0_) and 30 minutes post-operation (T_4_), the NS group exhibited a more significant decrease in Mean Arterial Pressure (MAP) and Heart Rate (HR) at various time intervals: 5 minutes after the skin incision (T_1_), 30 minutes post-incision (T_2_), and at the conclusion of the operation (T_3_), compared to the LS and HS groups(*P* < 0.05). Furthermore, the NS group exhibited a greater increase in levels of adrenaline (AD), noradrenaline (NE), endothelin (ET), C-reactive protein (CRP), tumor necrosis factor-alpha (TNF-α), and interleukin-6 (IL-6) at T_1_, T_2_, and T_3_, more so than the other two groups(*P* < 0.05). 24 hours after the surgery, patients in the LS group and HS group had significantly higher Montreal Cognitive Assessment (MoCA) scores than those in the NS group(*P* < 0.05). The LS and HS groups required lower doses of propofol, remifentanil, and sufentanil during surgery (*P* < 0.05), experienced shorter postoperative recovery times, and had lower incidences of nausea, vomiting, and respiratory depression compared to the NS group (*P* < 0.05).

**Conclusion:**

The administration of low-dose esketamine has been shown to be safe, effective, and dependable in the context of laparoscopic gallbladder surgery. It has the capacity to stabilize hemodynamic responses, ameliorate both stress and inflammatory reactions from surgery, and hastens anesthesia recovery. Furthermore, it fosters the restoration of postoperative cognitive function. Notably, when combined with nalbuphine, it exhibits opioid-sparing effects, reducing postoperative adverse outcomes.

**Trial registration:**

The trial is registered with the China Clinical Trials Registry Registration Number: ChiCTR2300067596. Retrospectively registered (date of registration: 12/01/2023).

## Background

Laparoscopic cholecystectomy, a common technique for treating gallbladder disease, is favored due to its relatively low complication rate, expedited postoperative recovery, minimized iatrogenic trauma, and decreased damage to the bile ducts [1]. However, the procedure necessitates the creation of a pneumoperitoneum, which can adversely affect the patient’s respiratory, circulatory, and neurological systems. If not properly managed, it can lead to cognitive impairment [2]. Postoperative cognitive dysfunction (POCD), a condition that can manifest in the weeks following non-vascular surgery, has been reported in 10–54% of cases. This dysfunction can be attributed to a variety of complex mechanisms, including surgical stimuli, anesthesia, medications, intraoperative hypotension, and inflammatory responses [3–5]. By employing appropriate anesthetic techniques and carefully selecting drugs, it is possible to mitigate the damage to the central nervous system caused by inflammatory factors and prevent the onset of postoperative cognitive dysfunction.

Opioids, as potent analgesics, are primarily employed for perioperative pain management due to their efficacy in suppressing the stress response and providing organ-protective effects, especially in instances of moderate to severe pain [6]. However, they are also associated with numerous adverse effects, including nausea, vomiting, constipation, central sensitization, and potential immunosuppression [7–10]. The magnitude of these adverse effects varies, wherein the extent of immunosuppressive activity emerges as a plausible discriminating factor. Some studies suggest that drugs with lower opioid receptor affinity exhibit less immunosuppressive activity, but this correlation may not hold true for all opioids. The immunosuppressive effects can be influenced by both pharmacodynamics and pharmacokinetics, as demonstrated by the differing properties of remifentanil, considered more immunosuppressive, and buprenorphine, which has virtually no immunosuppressive effects [11].

The concept of weak opioid anesthesia and analgesia presents a novel approach to perioperative pain management. Esketamine, the s-enantiomer of ketamine, is a novel anesthetic with sedative and analgesic properties. Unlike racemic ketamine, it has a three to four times higher affinity for NMDA receptors and exerts a lesser impact on the patient’s respiratory and circulatory systems [12, 13]. By stimulating the sympathetic nervous system and inhibiting NMDA receptors, it effectively increases blood pressure and induces bronchodilation. Consequently, this reduces intraoperative airway resistance and enhances pulmonary ventilation [14]. When administered in low continuous doses, esketamine effectively reduces postoperative sleep disturbances and pain incidence [15]. Moreover, its rapid antidepressant effects and its ability to suppress pro-inflammatory factors like IL-6 and TNF-α highlight its potential in mitigating postoperative cognitive dysfunction [16].

Due to the absence of specific guidelines for postoperative analgesic dosing of esketamine, studies have demonstrated that the efficacy of esketamine is twice that of ketamine [17]. This suggests that, for patients with acute pain who cannot tolerate high doses, low doses of esketamine (0.1–0.5 mg/kg·h) may be sufficient to achieve an analgesic effect. Consequently, esketamine could potentially serve as a potent adjunct to enhance anesthesia quality and mitigate complications. In the present study, we employed a low-dose esketamine regimen in conjunction with the fentanyl analog sufentanil, to facilitate mild opioid anesthesia during laparoscopic cholecystectomy. We aimed to evaluate its impact on hemodynamics, stress responses, inflammatory responses, and cognitive function.

## Method

### Ethical approval

**Fig. 1:**
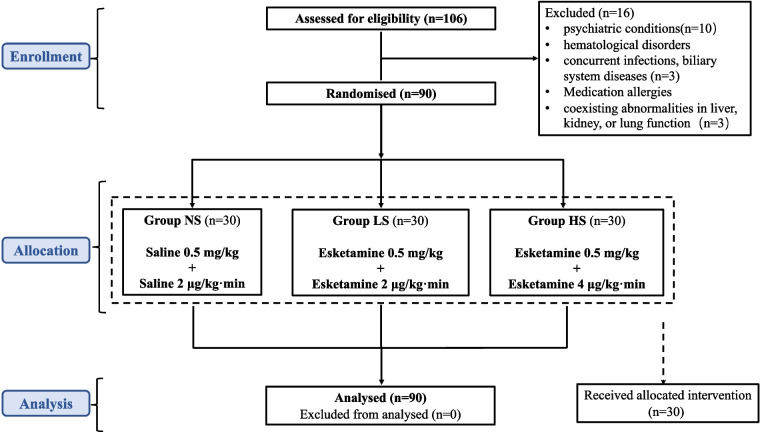
CONSORT patient enrolment diagram. CONSORT, Consolidated Standards of Reporting Trials

The study received ethical approval from the Human Research Ethics Committee of the Third Affiliated Hospital of Anhui Medical University on 28/03/2022 (Approval No. 2022–002, Principal Investigator: Prof. Dr. Yang). It was registered with the China Clinical Trial Registry (Registration No. ChiCTR2300067596, Principal Investigator: Yang Heng, Registration Date: 12/01/2023). All participants provided written informed consent to take part in the trial, and all procedures were conducted in accordance with the relevant guidelines and regulations. This manuscript adheres to the applicable Consolidated Standards of Reporting Trials (CONSORT) guidelines refer (Fig. 1).

### Patient cohort

Participants were enrolled in the study from February 2022 to January 2023, and the trial concluded after the final participant’s follow-up assessment. The study included individuals aged 18 to 65 years, scheduled for elective laparoscopic cholecystectomy, with an American Society of Anesthesiologists (ASA) classification of I or II, and demonstrating normal communication abilities. All participants provided informed consent before being included.

Exclusion criteria were comprehensive, encompassing patients with psychiatric conditions such as schizophrenia and delusional disorders, as well as those diagnosed with epilepsy. Individuals with known allergies to the study medications, concurrent infections, biliary system diseases, or coexisting abnormalities in liver, kidney, or lung function were also excluded. The study further eliminated patients with hematological disorders, including leukemia, aplastic anemia, malignant lymphoma, and myelodysplastic syndrome.

Additional exclusion criteria included patients with angina pectoris, malignant hypertension, elevated intracranial pressure, and mental disorders such as mood disorders, anxiety disorders, and obsessive-compulsive disorders. Patients with leukocyte disorders, severe coagulation irregularities, and profound anemia were also excluded from the study.

### Patient grouping and randomization

Participants were randomly allocated into one of three distinct groups: the LS group, the HS group, and the NS group, with each group encompassing 30 individuals. The LS and HS groups received a 0.5 mg/kg esketamine injection before the initial skin incision, followed by continuous esketamine infusions at 2 μg/kg·min and 4 μg/kg·min respectively. Conversely, the NS group received an intravenous dose of 0.5 mg/kg saline before the first skin incision, followed by a continuous saline infusion at a rate of 2 μg/kg·min. Other anesthesia procedures are identical for all three groups. The allocation to these groups was conducted using a randomized control chart, and to maintain the integrity of the blinding process, sealed opaque envelopes were employed. This ensured that both the data collectors and the study subjects remained unaware of the group assignments. Several anesthesiologists, who were blinded to the patient group allocations, participated in the study and strictly adhered to the prescribed experimental protocols for documenting intraoperative events. Furthermore, to enhance the scientific rigor of the study, a dedicated team of surgeons performed the procedures on all patients.

### Procedures

All patients were required to fast, abstaining from both food and liquids, for 6 hours before surgery. Upon admission, vital signs including electrocardiogram (ECG), HR, pulse oximetry, blood pressure (BP), and bispectral index (BIS) were monitored. Intravenous access was established in the upper limb, and a lactated Ringer solution was infused at a rate of 5 ml/kg·h.

Anesthesia was induced using a combination of intravenous medications: penehyclidine hydrochloride (0.5 mg), dexamethasone (10 mg), midazolam (0.03–0.06 mg/kg), sufentanil (0.4 μg/kg), etomidate (0.2–0.3 mg/kg), and cis-atracurium (0.1–0.3 mg/kg). Anesthesia maintenance was achieved through a continuous intravenous infusion of remifentanil (0.1–0.2 μg/kg·min) and propofol (0.1–0.2 mg/kg·min), with dosage adjustments made in accordance with the surgical procedure and BIS values.

Prior to anesthesia induction, electroencephalogram (EEG) dual-frequency index monitoring was performed on all patients using a standardized device and disposable sensor (Covidien, USA). The sensors were appropriately positioned on the forehead and temples. After applying pressure to the sensors for 5 seconds, the interface cable was connected, enabling accurate display of waveform and values on the anesthesia monitor. Cis-atracurium was administered at a dose of 0.02–0.03 mg/kg every 30 minutes.

Ventilation was maintained at a rate of 12–14 times/min, with an end-tidal carbon dioxide (P_ET_CO_2_) level of 35–45 mmHg, and an oxygen flow rate set at 2 L/min. The LS group received a single dose of 0.5 mg/kg esketamine prior to incision, followed by a continuous infusion of 2 μg/kg·min. The HS group received a similar initial dose of esketamine, but with a subsequent continuous infusion of 4 μg/kg·min. The NS group received a pre-incision dose of 0.5 mg/kg saline, followed by a continuous infusion at 2 μg/kg·min.

After completing the surgical procedure, all medications were discontinued. Patients were extubated and transferred to the ward once they regained consciousness. If patients reported a pain severity of ≥4 on a numerical rating scale (NRS) a single intravenous dose of 0.05 μg/kg sufentanil was administered. An NRS pain score of 1 or 2 was considered indicative of a satisfactory analgesic effect. However, the total amount of sufentanil administered over a three-hour period was not to exceed 0.1 μg/kg.

### Data collection

We obtained 3 ml of peripheral venous blood from the median cubital vein in the left elbow at various time intervals: prior to anesthesia (T_0_), 5 minutes post-incision (T_1_), 30 minutes post-incision (T_2_), immediately after the final incision closure (T_3_), and 30 minutes after the final incision closure (T_4_). The blood sample was promptly sealed and stored at 4 °C to maintain its purity. A designated individual transported these samples for testing within a four-hour window. The samples underwent centrifugation at 3000 rpm for 10 minutes, after which the supernatant was collected and assayed for AD and NE using a fluorometric assay, and ET using a radioimmunoassay. The detection kits utilized in this study were provided by Wuhan Purity Biotechnology Co.Ltd.

We employed an OTA-400 fully automatic biochemical analyzer, provided by Shenyang Wantai Medical Equipment Co. Ltd., to measure serum CRP levels, TNF-*α*, IL-6, and other inflammatory factors utilizing relevant enzyme-linked immunosorbent assays. We also documented the intraoperative and 24-hour postoperative sufentanil dosages, as well as the intraoperative isoproterenol and remifentanil dosages.

The anesthesiologist recorded the MAP and HR at the specified time points T_0_ to T_4_) during the surgery. These measurements, along with the MoCA scores, were recorded in a single logbook. The MoCA evaluated the patients’ cognitive function preoperatively and within 24 hours postoperatively. The MoCA scale, comprising eight items, assessed delayed recall, memory, abstract generalization, visuospatial and executive functions, attention, concentration, language, and calculation. The maximum score on the scale is 30, with scores below 10 indicating severe cognitive impairment, 10–17 indicating moderate cognitive impairment, 18–26 indicating mild cognitive impairment, and scores above 26 indicating no cognitive impairment [18].

We also documented the duration of the operation, the time of anesthesia, the time of awakening (first eye opening), and any post-operative adverse effects, such as nausea, vomiting, respiratory depression (tidal volume < 300 ml or respiratory rate < 10 min), excessive respiratory secretions, nightmares, hallucinations, and diplopia. The awakening time was defined as the period between the last administration of anesthetic drugs and the patient’s complete return to consciousness (Consciousness and orientation have been reestablished, with command-following motor functions and normalized muscle tone; the respiratory tract is unobstructed, and protective swallowing and cough reflexes are intact; the circulatory system is stable, with blood pressure and heart rate variations within 20% of preoperative levels, sustained for over 30 minutes, and a fundamentally normal ECG).

### Statistical analysis

Statistical analysis was conducted using SPSS software version 23.0 (IBM Corp, Armonk, NY, USA). Data with a normal distribution are presented as (*χ*^2^ ± *s*) values. Differences between groups were evaluated using independent samples *t*-tests, while paired *t*-tests were employed for within-group comparisons. Categorical data are expressed as *n* (%), and were analyzed using the *χ*^2^ test. The general linear repeated-measures analysis of variance was used to compare individual indices at multiple time points between the two groups. A *p*-value of less than 0.05 was considered statistically significant.

## Results

### Patient characteristics

90 eligible patients were included in the study and randomly assigned to one of the three groups. No significant differences were observed among the groups with respect to age, gender, ASA classification, and body mass index refer (Table 1).

**Table 1:** General information of patients who were included in the analysis

	NS	LS	HS
***n***	30	30	30
Age (yr; mean ± std)	39.3 ± 11.2	40.3 ± 10.3	39.5 ± 10.6
Sex (n; female/male)	15/15	14/16	16/14
ASA physical status (I/II)	16/14	15/15	14/16
BMI (kg/m^−2^)	22.5 ± 1.7	22.4 ± 1.6	22.8 ± 1.5

***n*** number of non-missing value: *BMI* body-mass index: *ASA* American Society of Anesthesiologists.

### Hemodynamics

**Fig. 2:**
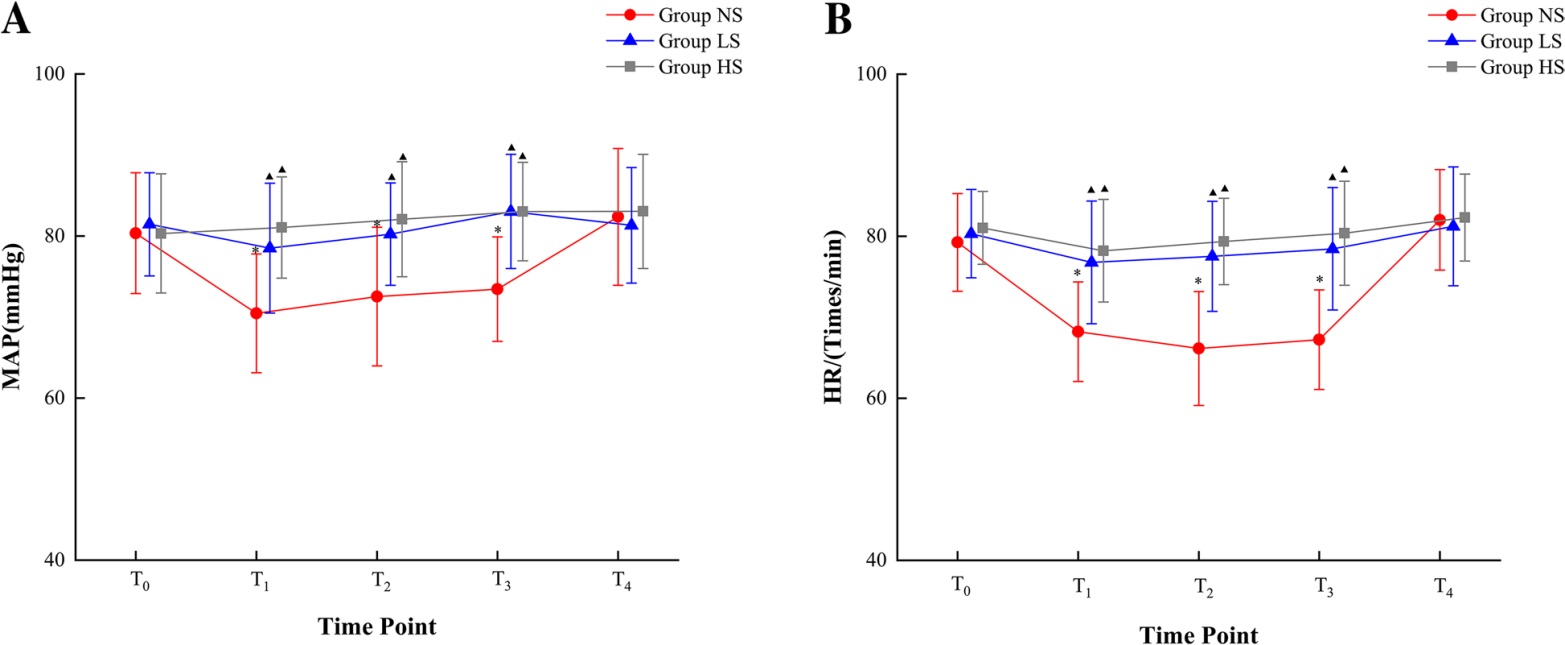
The graphs show the results of repeated measurements of hemodynamic parameters. **A** Mean arterial pressure (MAP); **B** Heart rate (HR). *: Indicates a significant difference when compared with the T_0_ and T_4_ time points within the same group, ^*^P < 0.05; **▲:** Indicates a significant difference when compared with time points in the NS group, ^**▲**^*P* < 0.05

At the T_0_ and T_4_ time points, there were no significant differences in MAP and HR between groups *P* > 0.05. However, in the NS group, MAP and HR were lower at T_1_, T_2_, and T_3_ compared to their values at T_0_ and T_4_. In contrast, at T_1_, T_2_, and T_3_, MAP and HR were higher in the LS and HS groups compared to T_0_ and T_4_ (*P* < 0.05) refer (Fig. 2).

### Intraoperative consumption of isoproterenol, remifentanil, and Sufentanil dosage in 24 hours

In both the LS and HS groups, a significant reduction was observed in the doses of isoproterenol, remifentanil, and sufentanil compared to the NS group refer (Table 2). However, there was no significant difference in dosage between the LS and HS groups (*P* > 0.05).

**Table 2:** Perioperative consumption of isoproterenol and remifentanil, intraoperative and 24-hour postoperative consumption of sufentanil

	NS	LS	HS
**n**	30	30	30
Isoproterenol (mg, mean ± std)	385.51 ± 10.82	320.53 ± 10.47^*^	319.66 ± 10.58^*^
Remifentanil (mg, mean ± std)	0.38 ± 0.08	0.30 ± 0.06^*^	0.29 ± 0.07^*^
Sufentanil (μg, mean ± std)	45.55 ± 3.45	29.61 ± 3.24^*^	28.92 ± 3.52^*^

* Indicates a significant difference when compared to the NS group, ^*^P < 0.05

### Stress response

**Fig. 3:**
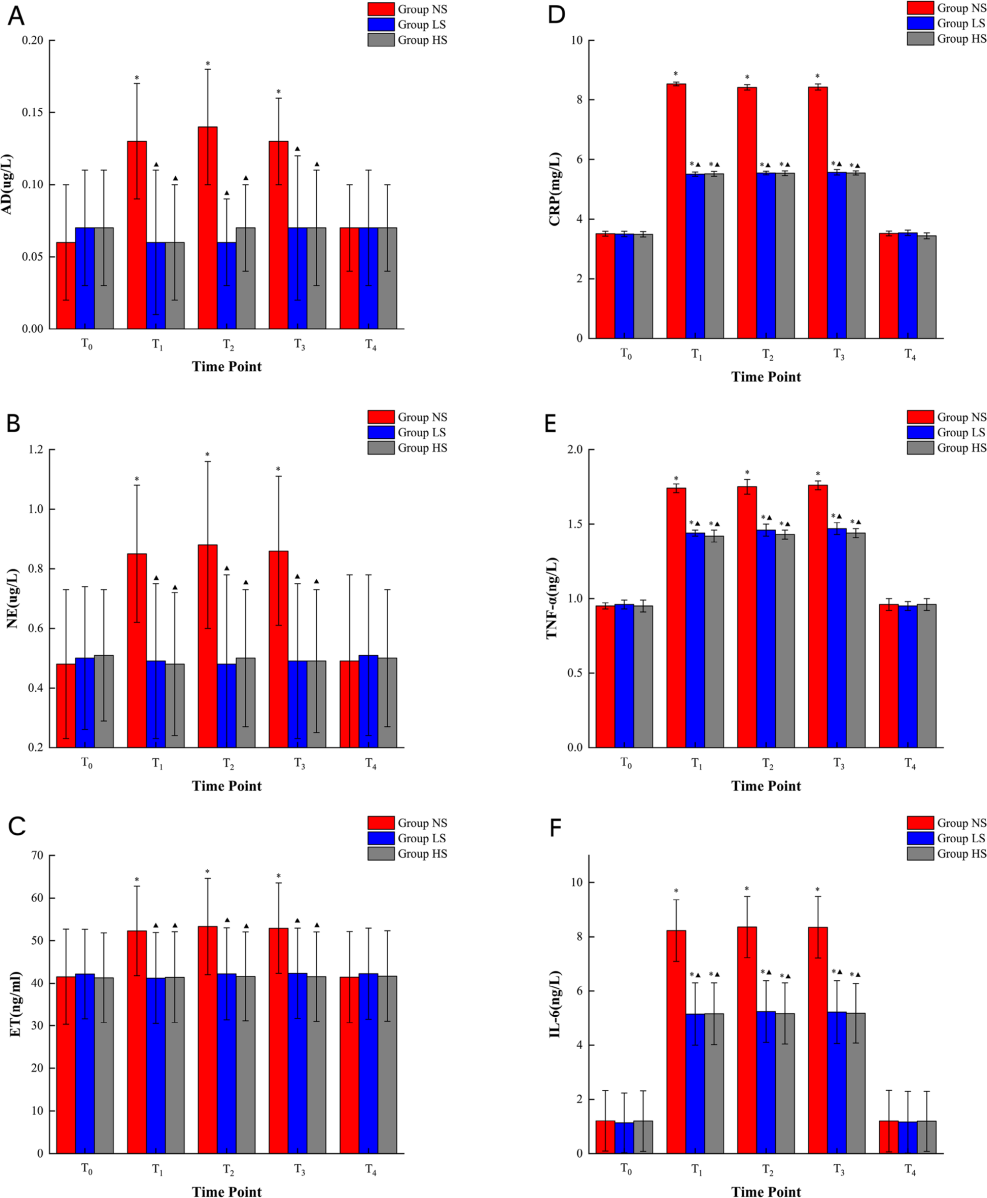
Results of repeated measurements of both stress and inflammatory response levels. **A** Adrenaline (AD); **B** Noradrenaline (NE); **C** Endothelin (ET); **D** C-reactive protein (CRP); **E** tumour necrosis factor-*α*(TNF-*α*); **F** interleukin-6(IL-6); *: Indicates a significant difference when compared with the T_0_ and T_4_ time points within the same group, ^*^P < 0.05; **▲**: Indicates a significant difference when compared with the NS group at the corresponding time point, ^**▲**^*P* < 0.05

Statistical analysis revealed no significant changes in the levels of blood AD, NE, and ET between the three groups at T_0_ and T_4_ time points (*P* > 0.05). However, in the NS group, levels of AD, NE, and ET at T_1_, T_2_, and T_3_ time points were significantly higher than those observed at T_0_ and T_4_. Conversely, a statistically significant reduction in the levels of AD, NE, and ET was observed in the LS and HS groups when compared to the NS group at T_1_, T_2_, and T_3_ time points(*P* < 0.05) refer (Fig. 3).

### Inflammatory response

There were no group differences in blood levels of CRP, TNF-*α*, and IL-6 at T_0_ and T_4_ (*P* > 0.05). However, in each group, levels of CRP, TNF-*α*, and IL-6 were significantly higher at T_1_, T_2_, and T_3_ compared to T_0_ and T_4_. We found that, at T_1_, T_2_, and T_3_, the LS and HS had lower levels of CRP, TNF-*α*, and IL-6 compared to the NS group (*P* < 0.05) refer (Fig. 3).

### Montreal cognitive assessment

There were no between-group differences in MoCA scores preoperatively (*P* > 0.05). MoCA scores decreased in all three groups at 24 hours postoperatively; however, the LS and HS groups had higher scores than the NS group (*P* < 0.05) refer (Table 3).

**Table 3:** Montreal Cognitive Assessment (MoCA) scores preoperatively and 24 hours postoperatively

	NS	LS	HS
***n***	30	30	30
Preoperative MoCA scores (mean ± std)	28.53 ± 1.45	28.72 ± 1.39	28.64 ± 1.42
MoCA score at 24 hours postoperatively (mean ± std)	22.79 ± 2.28^*^	26.80 ± 2.32^**#**^	26.75 ± 2.53^**#**^

*: Indicates a significant decrease from the preoperative score, *P < 0.05

**#**: Indicates a significant difference when compared to the NS group, ^**#**^*P* < 0.05

### Anesthesia indicators

There were no differences in operation time or anesthesia time between the three groups (*P* > 0.05), but the LS and HS groups had a faster awakening time than the NS group (*P* < 0.05) refer (Table 4).

**Table 4:** Perioperative-related indicators

	NS	LS	HS
***n***	30	30	30
Operating time (min, mean ± std)	50.22 ± 10.81	51.53 ± 10.42	50.83 ± 10.52
Anaesthesia time (min, mean ± std)	55.61 ± 11.25	56.31 ± 10.77	55.66 ± 10.91
Awakening time (min, mean ± std)	10.52 ± 3.43	5.81 ± 3.24^*^	5.55 ± 3.26^*^

* Indicates a significant difference when compared to the NS group, ^*^P < 0.05

### Post-operative adverse reactions

There were no differences between the three groups in terms of excessive respiratory secretion, nightmares, or hallucinations and double vision. The NS group experienced more nausea, vomiting, and respiratory depression than the LS and HS groups (*P* < 0.05) refer (Table 5).

**Table 5:** Post-operative adverse reactions

	NS	LS	HS
***n***	30	30	30
Nausea or vomiting (*n*/%)	5(16.67)	0(0.00)^*^	0(0.00)^*^
Respiratory depression (*n*/%)	4(13.33)	0(0.00)^*^	0(0.00)^*^
Hypersecretion (*n*/%)	0(0.00)	1(3.33)	1(3.33)
Nightmares (*n*/%)	0(0.00)	0(0.00)	1(3.33)
hallucinations or double vision (*n*/%)	0(0.00)	0(0.00)	1(3.33)

*Indicates a significant difference when compared to the NS group, ^*^P < 0.05

## Discussion

In this randomized controlled trial, we tested the effects of esketamine administration during laparoscopic cholecystectomy. In our study, low-dose esketamine proved effective in suppressing stress and inflammatory responses. Additionally, it induced mild excitatory sympathetic effects, which helped in preventing intraoperative hypotension. Furthermore, esketamine use resulted in opioid sparing and faster recovery from anesthesia, and also improved in 24-hour postoperative cognitive scores.

The minimally invasive aspects of LC technology have clear clinical benefits. However, the intraoperative establishment of a CO_2_ pneumoperitoneum adversely affects respiration, circulation, and postoperative cognitive function [19]. Pneumoperitoneum establishment elevates intra-abdominal pressure, which subsequently raises the diaphragm and increases intrathoracic pressure. This change leads to altered lung compliance [20, 21]. Additionally, the compression of abdominal vessels diminishes venous return to the central nervous system (CNS), inducing a state of ischemia and hypoxia, and is accompanied by an abundant production of reactive oxygen species [22]. Notably, inflammation within the central nervous system is one of the pivotal factors in the pathogenesis of postoperative cognitive dysfunction (POCD) [23–25]. Peripheral inflammatory mediators are significant contributors to this central nervous system inflammation [26, 27]. The presence of pneumoperitoneum intensifies the activation of the sympathetic nervous system triggered by surgical procedures, leading to a substantial release of norepinephrine and peripheral inflammatory cytokines [28]. Moreover, a rapid elevation in carbon dioxide partial pressure further aggravates this condition. Hypercapnia stimulates the overproduction of the cytokine IL-1β, which, through its interaction with microglial cells, triggers the release of additional inflammatory mediators [29]. This inflammatory cascade is intricately linked to deficits in hippocampus-dependent memory and consequently impairs cognitive functions [30–32]. LC necessitates meticulous management of intraoperative analgesia and inotropic relaxation, alongside real-time adjustments to the physiological disturbances that CO_2_ pneumoperitoneum causes on respiration and circulation. This ensures that seamless hemodynamic stability and rapid postoperative awakening are attained. We observed that low-dose esketamine and high-dose esketamine groups exhibited more stable arterial pressure and heart rate, and intraoperative epinephrine, norepinephrine, and endothelin levels were significantly lower compared to the control group. These findings are in agreement with previous work showing that the effects of esketamine are dose-dependent and that it can directly inhibit the stress response by stimulating the sympathetic nervous system and indirectly stimulate cardiovascular function resulting in an increased heart rate, cardiac output, and blood pressure, yet with minimal effects on the peripheral vasculature, thus indicating a favorable profile for perioperative management [33].

Compared to the control group, both the low-dose and high-dose esketamine groups exhibited reduced 24 hours consumption of sufentanil, intraoperative remifentanil, and propofol along with faster postoperative arousal times. Rapid arousal mainly stems from the redistribution and metabolization of the sedative agent [34]; these results are in line with the results of previous studies [35]. In vivo, esketamine is mainly converted to nor-esketamine by hepatic microsomal enzymes. Nor-esketamine is pharmacologically active, with an anesthetic potency approximately 1/5 to 1/3 that of esketamine. Its elimination half-life is longer, which accounts for the prolonged analgesic effect of esketamine, even after patients awaken from anesthesia [12]. In addition, esketamine blocks the glutamate NMDA, inhibiting possible hypersensitization changes in the dorsal horn of the spinal cord. Esketamine also affects peripheral NMDA receptors to exert analgesic sedation, inhibit opioid nociceptive hyperalgesia, reduce opioid consumption, and enhance postoperative incisional analgesia [36]. The activation of potassium channels by calcium ions in microglia may be one of the mechanisms by which esketamine exerts its analgesic effects [37]. Low doses of esketamine not only reduce the severity of the choking response during recovery from general anesthesia but also ensure a smoother arousal process with fewer adverse effects, resulting in a more comfortable awakening period for the patient [38]. These findings are consistent with the opioid-sparing effects found in our cohort.

When examining inflammatory markers, we found that CRP, TNF-*α*, and IL-6 levels were lower at T_1_, T_2_, and T_3_ in the low-dose and high-dose esketamine groups compared to the control group. It has been demonstrated that ketamine can inhibit the production of various pro-inflammatory cytokines and the lipopolysaccharide (LPS)-induced inflammatory response in astrocytes and microglia. Employing the HMC3 human microglial cell line, ketamine and its active metabolites have been shown to be involved in regulation of the type I interferon pathway. This modulation is mediated through the activation of signal transducer and activator of transcription 3 (STAT_3_), which plays a crucial role in orchestrating the immune response [39]. It has been found that esketamine can be used in combination with hypnotic agents for general anesthesia induction and maintenance. Esketamine, when administered at a dosage of 0.25–1 mg/kg, has the potential to exhibit anti-inflammatory effects. Specifically, it can effectively suppress the release of inflammatory cells that are stimulated by oxygen free radicals, thereby mitigating the overall inflammatory response. When used in conjunction with opioids, the dosage of esketamine can be judiciously reduced while maintaining therapeutic efficacy [40]. In addition, when coadministered, esketamine reduces the distribution and clearance of propofol, increases its plasma concentrations, inhibits pressure receptor sensitivity, and stabilizes patient hemodynamics [41]. Previous studies have found that esketamine can reduce the levels of interleukin-10 in the plasma of patients undergoing extracorporeal circulation 1 day postoperatively, demonstrating corresponding anti-inflammatory effects. This is largely consistent with the results of our current research [42]. In our study, patients in the esketamine group had higher MoCA cognitive function scores than controls, possibly due to the unique efficacy of esketamine’s antidepressant properties. Postoperative cognitive dysfunction (POCD), potentially induced by anesthesia, can be triggered by various factors. Factors contributing to POCD include disruptions in calcium homeostasis and systemic inflammatory responses triggered by surgical trauma. Additionally, the inhibition of age-sensitive neuronal stem cell function and the acceleration of ongoing endogenous neurodegenerative processes can play a role. These factors may exert direct or indirect adverse effects on cognitive function. Esketamine has been shown to reduce cognitive impairment by stimulating the activity of the interferon GRNES/TANK-binding kinase 1(TBK1) signaling pathway. This activity impedes trauma-induced chronic neuroinflammation, blood-brain barrier disruption, oxidative stress, and microglia activation [40, 43–47]. In our study, we observed that postoperative nausea and vomiting and respiratory depression were also significantly less common than in the control group. Adverse effects such as nightmares and diplopia were largely absent, probably due to the use of esketamine with weak opioid anesthesia to reduce opioid adverse effects, and the intraoperative combination of propofol and midazolam to reduce the psychiatric side effects of esketamine. However, in the high-dose group, adverse reactions such as excessive respiratory secretion, nightmares and diplopia occurred in individual cases each accounting for 3.33%.

### Limitations

This study has some noteworthy limitations. First, postoperative cognitive impairment can persist for several weeks. To gain a more nuanced understanding of the effects of esketamine, prolonged follow-up assessments incorporating continuous cognitive profiling are required. Second, the current investigation provides findings in the context of the specific surgical procedure under study. Extrapolation of these findings to other laparoscopic surgeries calls for a cautious approach. Consequently, there is a need for future studies to broaden the scope of this research to properly test the generalizability of our findings.

## Conclusions

A preoperative administration of 0.5 mg/kg of esketamine followed by an intraoperative maintenance dose of 2 μg/kg·min was found to be effective in maintaining hemodynamic stability and reducing surgical stress and inflammation in patients undergoing laparoscopic cholecystectomy. This regimen resulted in a shorter post-anesthesia arousal time, reduced opioid consumption, fewer postoperative adverse effects, and improved cognitive recovery. Esketamine, with its weak opioid properties, was found to be safe, effective, and reliable for laparoscopic cholecystectomy, and may have clinical value in this setting.

## Data Availability

The datasets generated and analysed during the current study are not publicly available due to institutional restrictions but are available from the corresponding author upon request. The corresponding author can be reached at yangh999@yeah.net.
